# Influence of weight perception on the evolution of body composition of adolescents under obesity treatment

**DOI:** 10.1007/s00431-025-06737-x

**Published:** 2026-01-23

**Authors:** Helena Fonseca, Rita Leiria, António Videira-Silva

**Affiliations:** 1 WHO Collaborating Centre for Adolescent Medicine and Training, Clínica Universitária de Pediatria, Faculdade de Medicina, Universidade de Lisboa, Lisbon, Portugal; 2 Pediatric Obesity Clinic, Department of Pediatrics, Hospital de Santa Maria, Lisbon, Portugal; 3 Faculdade de Medicina, Universidade de Lisboa, Lisbon, Portugal; 4 CIDEFES (Centro de Investigação Em Desporto, Educação Física, Exercício E Saúde), Universidade Lusófona, Lisbon, Portugal; 5 CIFI2D (Research Centre in Physical Activity, Health and Leisure), Universidade Do Porto, Porto, Portugal

**Keywords:** Adolescents, Obesity, Body composition, Self-perception, Body self-image

## Abstract

Obesity is a public health challenge, especially in adolescence, a stage characterized by physiological and behavioral changes that may influence energy balance, lifestyle choices, and long-term health trajectories. Although self-image place a significant role on weight perception, there is no consensus on the impact of self-perceived weight on actual weight development. While some studies suggest that underestimating weight may contribute to subsequent weight gain, others suggest the contrary. The aim of this study was to analyze the influence of weight perception on the evolution of body composition in adolescents followed in an Adolescent Obesity Clinic. An exploratory, longitudinal, retrospective study was carried out, analyzing data from 131 adolescents with obesity (BMI Z-Score ≥ 2.0), with valid data from at least two obesity appointments. 44.3% of the participants had a correct perception of their weight evolution; 41.4% perceived an increase, with real weight loss and 7.1% reported having no perception at all of their weight evolution. Lack of perception of weight evolution was associated with an increase in hip circumference (β = 1.021; 95%CI: 0.791 to 1.318; R^2^ = 31.6%; *p* = .012). The correct perception of weight gain, compared to the wrong perception of weight loss, was associated with a greater increase in BMI (respectively, β = 5.833; 95%CI: 2.223; 15.304; R^2^ = 67.2%; *p* < .001; β = .131; 95%CI: 0.003; 0.292; R^2^ = 85%; *p* < .001).

*Conclusion*: Accurate perception of body weight changes may influence positively body composition in adolescents with obesity.

**Table:** 

**What is Known:** • *Adolescence is a critical developmental period for obesity, during which body image and weight perception play a central role in health behaviors and treatment engagement.* • *Weight status misperception is common among adolescents with obesity and may act as a barrier to effective weight management; however, data focusing specifically on adolescents under obesity treatment are limited.*
**What is New:** • *In adolescents with obesity followed in a specialized clinic, inaccurate and lack of perception of weight change is highly prevalent, even under regular clinical monitoring, being associated with unfavorable changes in body composition.* • *Accurate perception of weight change emerges as a relevant factor linked to body composition evolution, underscoring the clinical importance of assessing and addressing weight perception during adolescent obesity treatment.*

**What is Known:**

• *Adolescence is a critical developmental period for obesity, during which body image and weight perception play a central role in health behaviors and treatment engagement.*

• *Weight status misperception is common among adolescents with obesity and may act as a barrier to effective weight management; however, data focusing specifically on adolescents under obesity treatment are limited.*

**What is New:**

• *In adolescents with obesity followed in a specialized clinic, inaccurate and lack of perception of weight change is highly prevalent, even under regular clinical monitoring, being associated with unfavorable changes in body composition.*

• *Accurate perception of weight change emerges as a relevant factor linked to body composition evolution, underscoring the clinical importance of assessing and addressing weight perception during adolescent obesity treatment.*

## Introduction

Obesity is a public health challenge of the twenty-first century, negatively affecting all human organs and systems [1–3]. Adolescence is a critical period in human development, marked by biological, cognitive, psychological and social transformations [4, 5], being a period of vulnerability to the development of eating disorders, including obesity [6].

An important component of self-assessment and hetero-assessment of body weight is exposure to the bodies of others [7, 8]. As the prevalence of obesity has increased, exposure to larger bodies have become more frequent within the population [9]. According to the normalization theory, increased exposure to larger bodies and consequent normalization of higher body weights may lead many individuals who meet body mass index (BMI) criteria for obesity to no longer recognize or classify themselves as having obesity [10].

The transtheoretical model for change indicates that awareness of a condition is necessary for its management and treatment [11]. Thus, if a large proportion of individuals with obesity do not identify themselves as having obesity, this can constitute a barrier to weight control [12], perpetuating obesity.

Studies in adults, however, suggest the contrary. Self-identification as having obesity, whether accurate or not, may induce psychological distress, which may negatively impact lifestyle behavioral change and weight, in turn [13].

The inconsistency found in the literature on the subject, as well as the lack of evidence in the adolescent population, highlights the need for more studies.

This study aimed to analyze the influence of weight perception on the evolution of body composition in adolescents with obesity, followed in a Adolescent Obesity Clinic.

## Methods

### Study design

This exploratory study was designed as a retrospective longitudinal study, using a non-probabilistic, non-intentional, convenience sampling method.

### Sample

Inclusion criteria included adolescents (12 to 18 years old), followed at the Adolescent Obesity Clinic, Pediatrics Department, ULSSM, with first appointment between January 2023 and August 2024. Only participants with valid data on the main variables of interest, in at least two appointments (first-time appointment and follow-up), were included in the analysis. Adolescents with intellectual development disorders with impaired comprehension were excluded.

### Procedure

This study was performed in line with the principles of the Declaration of Helsinki. Approval was granted by the Ethics Committee of the Faculty of Medicine, University of Lisbon (January 30, 2025/No 332/24).

The clinical records of each participant were consulted to collect relevant information. After collecting the information, the confidentiality of the data collected was ensured by assigning a random numerical identifier code to each participant.

The data collected was recorded in an Excel document, access to which was password-protected.

### Variables and measuring instruments

Sociodemographic and contextual variables were collected by clinical interview. Age, ethnicity, nutritional status of the father and mother, age at onset of obesity, duration of obesity, and presence of comorbidities were determined and collected during the medical appointment. The time between the first and follow-up appointments was calculated.

Anthropometric assessments, including height (cm) and weight (kg), waist circumference (cm), and hip circumference (cm) were measured using standard procedures by the same researcher. BMI and BMI z-score were further calculated using the “WHO AnthroPlus” software.

Body composition, including absolute fat and muscle mass (kg) was assesed using a bioelectrical impedance scale (InBody 230, Seoul, Korea), and the relative values were calculated (i.e., fat mass % and muscle mass %).

Duration of structured and total weekly physical activity (min/day) was assessed at the clinical exercise appointment by interview [14].

In order to characterize perceived weight gain, participants were asked whether they perceived weight loss, maintenance or gain, or whether they showed no perception of their weight evolution between the two study periods. For those who were aware of their weight change, they were also asked about their objective weight perception, which is the weight they thought they had at the time.

### Statistical analysis

The data were analyzed using IBM-SPSS software (version 29.0), considering statistical significance for *p* < 0.05. The normality of the variables was assessed using the Shapiro–Wilk test and direct observation. To compare quantitative variables between sexes, the t-test (normal distribution) and the Mann–Whitney test (non-normal distribution) were used; for categorical variables, the chi-square test was used. The t-test was also applied to compare participants assessed at two time points with those excluded from the longitudinal analysis, as well as to assess temporal evolution and differences between sexes in anthropometric measurements, body composition, and physical activity. Logistic regressions analyzed the association between subjective perception of weight change and actual changes in measurements. To assess the temporal evolution of these variables according to weight perception (actual or subjective), the ANOVA test was used, followed by the Bonferroni test when significant differences were identified.

## Results

### Sample characteristics

The initial sample consisted of 165 participants with valid data, including 83 girls (50.3%) and 82 boys (49.7%). A statistically significant difference was found between sexes in the presence of psychopathology, with a lower frequency in girls compared to boys (MD: −16.7%, *p* = 0.019). In addition, girls had statistically lower height (MD: −6.1 cm, *p* < 0.001) and weight (MD: −8.0 kg, *p* = 0.007) than boys (Table 1.).

**Table 1.** Baseline characteristics of the participants

Variables		Girls (*n* = 83)	Boys (*n* = 82)		Total (*n* = 165)
		*n* (%)		*p*-value	*n* (%)
Ethnicity (Caucasian)		54 (65.1%)	56 (68.3%)	.297^a^	109 (66.1%)
Nutritional Status Father	Pre-obesity	9 (10.8%)	8 (9.8%)	.805^a^	17 (10.3%)
	Obesity	9 (10.8%)	12 (14.6%)		21 (12.7%)
Mother's nutritional status	Pre-obesity	14 (16.9%)	9 (11.0%)	.319^a^	23 (13.9%)
	Obesity	22 (26.5%)	21 (25.6%)		43 (26.0%)
Psychopathology		22 (26.5%)	36 (43.2%)	**.019**^**a**^	58 (35.2%)
Cardiometabolic pathology		19 (22.9%)	19 (23.2%)	.966^a^	38 (23.0%)
Immunoallergological pathology		16 (19.3%)	16 (19.5%)	.970^a^	32 (10.4%)
		**Mean ± SD**			**Mean ± SD**
Age (years)		14..6 ± 1..9	14.2 ± 2.3	.162^b^	14.4 ± 2.2
Onset of obesity (months)		90 (120)	96 (72)	.846^c^	96 (90)
Obesity duration (months)		82..0 (110.0)	75.5 (77.0)	.413^c^	76.0 (92.0)
Time between appointments (months)		6..0 (4.5)	6.0 (3.3)	.921^c^	6.0 (3.0)
Height (cm)		160.3 ± 7.1	166.4 ± 9.7	**.000**^**b**^	163.4 ± 9.0
Weight (kg)		82.4 (20.6)	90.4 (34.7)	**.007**^**c**^	84.4 (27.5)
BMI (kg/m2)		31.75 (6.13)	32.04 (9.22)	.277^c^	31.95 (7.25)
BMI Z-Score		2.67 (0.75)	2.91 (1.07)	**.003**^**c**^	2.76 (0.99)
Waist circumference (cm)		96.6 (13.5)	104.3 (23.2)	**.000**^**c**^	99.2 (16.6)
Hip perimeter (cm)		114.1 (15.4)	114.6 (18.9)	.307^c^	114.1 (17.8)
Absolute fat mass (Kg)		37.4 (14.6)	36.75 (20.3)	.763^c^	37.4 (16.5)
Relative fat mass (%)		44.5 ± 5.7	41.2 ± 8.8	**.007**^**b**^	42.8 ± 7.6
Absolute muscle mass (Kg)		24.2 (6.2)	33.0 (11.7)	**.000**^**c**^	27.1 (10.7)
Relative muscle mass (%)		30.6 (5.1)	32.7 (6.1)	**.003**^**c**^	31.1 (5.1)
Structured physical activity (min)		150 (60)	150 (136)	.839^c^	150 (86)
Total physical activity (min)		205 (214)	197 (220)	.781^c^	199 (225)

*SD* Standard Deviation, *BMI* Body Mass Index

^a, b, c^ Differences between groups analysed with Chi-square test. Independent Sample *t*-test and Mann–Whitney U-

test. respectively. For the Mann–Whitney U-test. median values (interquartile range) are shown

Values in bold indicate statistically significant values (*p-*value ≤.05)

At baseline, girls showed a lower BMI z-score (MD: −0.24, *p* = 0.003), waist circumference (MD: −7.7 cm, *p* < 0.001), and absolute and relative muscle mass (DM: −8.8 kg, *p* < 0.001; DM: −2.1%, *p* = 0.003) compared to boys. On the other hand, relative fat mass was statistically higher in girls (MD: 3.3%, *p* = 0.007) (Table 1.).

There were no statistically significant differences between the sexes in the other study variables.

Of the 165 participants suitable for inclusion, 131 presented valid longitudinal data, and thus were included in the longitudinal analysis (retention rate of 79.4%). No statistically significant differences were found between the baseline and the longitudinal sample (Table 2).

**Table 2:** Differences between baseline and longitudinal samples

Variables	Included (*n* = 131)	Excluded (*n* = 34)	*p-value*
Girls (n. %)	65 (49.6)	18 (52.9)	.730^a^
Age (years)	14.4 ± 0.2	14.6 ± 0.3	.594
Obesity duration (months)	87.9 ± 5.1	97.5 ± 11.0	.409
Height (cm)	163.1 ± 0.8	164.2 ± 1.6	.548
Weight (kg)	89.1 ± 2.0	89.9 ± 3.5	.856
BMI (kg/m2)	33.24 ± 0.64	33.10 ± 0.92	.916
BMI Z-Score	3.04 ± 0.12	2.89 ± 0.13	.527
Waist circumference (cm)	127.7 ± 15.3	136.3 ± 34.6	.804
Hip perimeter (cm)	149.1 ± 17.3	149.8 ± 34.0	.985
Absolute fat mass (Kg)	39.1 ± 1.4	39.3 ± 2.3	.946
Relative fat mass (%)	42.9 ± 0.7	42.4 ± 1.3	.769
Absolute muscle mass (Kg)	29.8 ± 2.0	28.9 ± 1.2	.810
Relative muscle mass (%)	34.0 ± 2.4	31.7 ± 0.7	.637
Structured physical activity (min)	164.6 ± 10.1	182.2 ± 22.1	.449
Total physical activity (min)	232.9 ± 13.8	242.2 ± 31.1	.769

*BMI* Body Mass Index

^a^ Differences analysed using the Chi-squared test

Values in bold indicate statistically significant values (*p*-value ≤.05)

### Longitudinal changes and gender differences in anthropometric values, body composition and physical activity

Between the first and second assessment moments, there was a statistically significant increase in height in both girls (∆1.0 cm, *p* < 0.001) and boys (∆2.2 cm, *p* < 0.001), with a statistical difference between sexes (MD: 1.26 cm, 95%CI −1.96 to −0.57, *p* < 0.001). On the other hand, statistically significant reductions were found in BMI z-score, in both girls (∆ −0.14 cm, *p* = 0.002) and boys (∆ −0.29 cm, *p* = 0.011), and in relative fat mass, but just in boys (∆ −2.3%, *p* = 0.012). No statistical significance between the sexes were found in these variables (Table 3).

**Table 3:** Longitudinal changes and gender differences in anthropometric values. body composition and physical activity

	Girls (*n* = 83)			Boys (*n* = 82)			Time*Gender (*n* = 165)	
*Variáveis*	*Initial*	*Final*	*p-value*	*Initial*	*Final*	*p-value*	*MD* (IC 95%)	*p-value*
Height (cm)	160.0 ± 6.8	161.0 ± 7.3	** <.001**	166.2 ± 9.3	168.4 ± 8.4	** <.001**	−1.26 (−1.96 a −0.57)	** <.001**
Weight (kg)	83.5 ± 18.8	83.7 ± 18.7	.694	94.7 ± 26.2	96.2 ± 26.2	.052	−1.22 (−3.13 a 0.69)	.208
BMI (kg/m^2^)	32.48 ± 6.72	32.28 ± 6.85	.404	33.99 ± 7.80	33.65 ± 7.93	.208	0.13 (−0.56 a 0.83)	.704
BMI Z-Score	2.80 ± 1.01	2.66 ± 1.05	**.002**	3.28 ± 1.54	2.99 ± 1.17	**.011**	0.15 (−0.09 a 0.39)	.218
Waist circumference (cm)	95.7 ± 10.1	98.2 ± 14.1	.187	133.8 ± 153.9	132.5 ± 154.2	.973	3.84 (−77.89 a 85.56)	.926
Hip perimeter (cm)	113. 2 ± 10.8	142.5 ± 162.3	.324	169.2 ± 214.5	140.5 ± 154.8	.549	57.93 (−55.85 a 171.71)	.313
Absolute Fat Mass (kg)	36.9 ± 11.4	35.8 ± 12.7	.129	39.3 ± 18.0	38.0 ± 17.3	.273	0.12 (−2.56 a 2.80)	.927
Relative Fat Mass (%)	44.0 ± 6.3	42.6 ± 8.9	.062	41.3 ± 8.9	39.0 ± 9.2	**.012**	0.90 (−1.39 a 3.19)	.435
Absolute Muscle Mass (Kg)	25.2 ± 4.3	25.9 ± 5.8	.247	34.0 ± 31.1	31.7 ± 7.8	.575	3.08 (−5.55 a 11.71)	.480
Relative Muscle Mass (%)	30.8 ± 3.5	31.6 ± 5.4	.220	38.0 ± 39.6	34.1 ± 5.4	.480	4.69 (−6.66 a 16.03)	.415
Structured Physical Activity (min)	156.4 ± 101.0	158.6 ± 117.8	.893	174.4 ± 132.8	186.8 ± 182.3	.556	−10.20 (−63.04 a 42.64)	.703
Total Physical Activity (min)	221.3 ± 142.9	204.1 ± 164.6	.463	242.3 ± 175.1	232.3 ± 212.3	.698	−7.25 (−75.84 a 61.34)	.835

*MD* Mean difference, *CI* Confidence interval, *BMI* Body mass index

Values in bold indicate statistically significant values (*p -value* ≤.05)

No statistically significant longitudinal changes were found, as shown in Table 3.

### Perceived weight change

The participants' perceived weight change is shown in Table 4. Eight participants (7.1%) had no perception of their weight evolution, while eight participants (7.1%) also had a perception of weight maintenance. 53 participants (47.3%) perceived an increase in weight, while 43 participants (38.4%) perceived a decrease. There was no statistically significant difference between sexes in terms of perceived weight gain (Table 4).

**Table 4:** Perceived weight evolution

Perceived Weight Gain	Girls (*n* = 56)	Boys (*n* = 56)	*p-value*	Total (*n* = 112)
No perception	5 (4.5%)	3 (2.7%)	.875 ^a^	8 (7.1%)
Perception of maintenance	4 (3.6%)	4 (3.6%)		8 (7.1%)
Perception of weight loss	22 (19.6%)	21 (18.8%)		43 (38.4%)
Perception of weight gain	25 (22.3%)	28 (25.0%)		53 (47.3%)
Perceived vs. Actual Evolution	Girls (*n* =51)	Boys (*n* = 53)	*p-value*	Total (*n*= 104)
Correct perception of weight loss	2 (1.9%)	2 (1.9%)	.767 ^a^	4 (3.9%)
Correct perception of weight gain	21 (20.2%)	21 (20.2%)		42 (40.4%)
Wrong perception of weight loss	19 (18.3%)	24 (23.1%)		43 (41.4%)
Wrong perception of weight gain	9 (8.7%)	6 (5.8%)		15 (14.4%)

^a^ Differences analysed using the Chi-squared test

Values in bold indicate statistically significant values (*p-value* ≤.05)

Forty-six participants (44.3%) accurately perceived their weight gain, four participants (3.9%) accurately perceiving weight loss and 42 participants (40.4%) accurately perceiving weight gain. 58 participants (55.8%) had an inaccurate perception of their weight evolution, with 43 (41.4%) having an inaccurate perception of weight gain and 15 (14.4%) an inaccurate perception of weight loss, with no statistically significant differences between the sexes (Table 4).

### Associations between perception of weight gain and variation in anthropometrics and physical activity

Table 5 shows multiple regression models with the association between perception of weight change (gain, loss or no perception) and variation in anthropometrics and physical activity, with only the statistically significant variables represented.

**Table 5:** Multiple regression models using variation in hip circumference. variation in body mass index and variation in structured physical activity as dependent variables

	Δ Hip circumference				Δ BMI				Δ Structured Physical Activity			
Perception of weight gain	β	*IC (95%)*	*R2*	*p*	β	*IC (95%)*	*R2*	*p*	β	IC (95%)	*R2*	*p*
Model 1 | No perception of weight gain	1.021	(0.791; 1.318)	.316	**.012**								
Model 2 | Perceived weight loss					.395	(0.231; 0.678)	.401	**<.001**	.994	(0.989; 1.000)	.500	**<.001**
Model 3| Perceived weight gain					2.274	(1.425; 3.629)	.377	**<.001**				
Perceived versus actual weight gain												
Model 4| Correct perception of weight gain					5.833	(2.223; 15. 304)	.672	**<.001**				
Model 5| Misperception of weight loss					.131	(0.003; 0.292)	.850	**<.001**				

*BMI* Body Mass Index; *CI* Confidence Interval

Values in bold indicate statistically significant values (p -value ≤.05)

Variables excluded:

Model 1 – ΔHeight, ΔWeight, ΔBMI, ΔZ-Score BMI, ΔHeight Circumference, ΔAbsolute Fat Mass, ΔRelative Fat Mass, ΔStructured Physical Activity, and ΔTotal Physical

Activity

Model 2 – ΔHeight, ΔWeight, Δz-score BMI, ΔHip Circumference, ΔWaist Circumference, ΔAbsolute Fat Mass, ΔRelative Fat Mass, ΔAbsolute Muscle Mass, ΔRelative Muscle Mass, and ΔTotal Physical Activity

Model 3—ΔHeight, ΔWeight, Δz-score BMI, ΔHip Circumference, ΔWaist Circumference, ΔAbsolute Fat Mass, ΔRelative Fat Mass, ΔAbsolute Muscle Mass, ΔRelative Muscle Mass, ΔStructured Physical Activity, and ΔTotal Physical Activity

Model 4—ΔHeight, ΔWeight, Δz-score BMI, ΔHip Circumference, ΔWaist Circumference, ΔAbsolute Fat Mass, ΔRelative Fat Mass, ΔAbsolute Muscle Mass, ΔRelative Muscle Mass, ΔStructured Physical Activity, and ΔTotal Physical Activity

Model 5—ΔHeight, ΔWeight, Δz-score BMI, ΔHip Circumference, ΔWaist Circumference, ΔAbsolute Fat Mass, ΔRelative Fat Mass, ΔAbsolute Muscle Mass, ΔRelative Muscle Mass, ΔStructured Physical Activity, and ΔTotal Physical Activity

Lack of perception of weight gain was associated with a significant increase in hip circumference (β = 1.021; 95%CI: 0.791 to 1.318; R^2^ = 31.6%; *p* = 0.012). Perceived weight loss was associated with an actual increase in BMI (β = 0.395; 95%CI: 0.231; 0.678; R^2^ = 40.1%; *p* < 0.001) and a longer duration of structured physical activity (β = 0.994; 95%CI: 0.989; 1.000; R^2^ = 50%; *p* < 0.001). It was also found that the perception of weight gain was associated with a significant increase in BMI (β = 2.274; 95%CI: 1.425; 3.629; R^2^ = 31.6%; *p* < 0.001) (Table 5).

With regard to the perception versus reality of weight gain, participants who correctly perceived weight gain showed a strong association with a real increase in BMI (β = 5.833; 95%CI: 2.223; 15.304; R^2^ = 67.2%; *p* < 0.001). The same was true in the participants with an erroneous perception of real weight loss (β = 0.131; 95%CI: 0.003; 0.292; R^2^ = 85%, *p* < 0.001) (Table 5).

### Longitudinal changes in anthropometrics, body composition and physical activity according to perceived weight gain

Between the groups with no perception of ponderal evolution and those with a perception of ponderal loss, there was a ponderal reduction, with a statistical difference between the groups (DM: 0.9 kg, *p* = 0.020). The groups with perceived weight loss and perceived weight gain also showed statistically significant differences in weight gain (DM: 6.2 kg, *p* < 0.001). Between the groups with perceived weight loss and perceived weight gain, there were also statistically significant differences in BMI variation (∆0.82 kg/m^2^, *p* < 0.001) (Table 6).

**Table 6:** Longitudinal changes in anthropometrics. body composition and physical activity according to perceived weight gain

*Variables*	No perception	Perceived weight maintenance	Perceived weight loss	Perceived weight gain
∆Height (cm)	0.8 ± 0.3	1.0 ± 0.2	1.5 ± 0.3	2.1 ± 0.3
∆Weight (kg)	**−1.6 ± 0.2**^**(1)**^	1.0 ± 1.2	**−2.5 ± 0.7 **^**(2)**^	**3.7 ± 0.7**^**(1) (2)**^
∆BMI (kg/m^2^)	0.96 ± 0.44	−0.08 ± 0.34	**−1.47 ± 0.32 **^**(2)**^	**0.65 ± 0.24 **^**(2)**^
∆BMI Z-Score	**−1.03 ± 0.84 **^**(1)**^	−0.11 ± 0.06	−0.33 ± 0.06	**−0.05 ± 0.04 **^**(1)**^
∆Waist circumference (cm)	−5.5 ± 1.4	0.4 ± 2.2	−56.4 ± 53.6	3.3 ± 1.8
∆Hip perimeter (cm)	214.1 ± 220.3	−2.6 ± 4.4	−55.0 ± 52.7	−26.9 ± 288.1
∆Absolute fat mass (kg)	−2.0 ± 1.2	−3.4 ± 4.3	**−4.0 ± 1.5 **^**(2)**^	**1.1 ± 0.7 **^**(2)**^
∆Relative fat mass (%)	−1.0 ± 0.7	−5.3 ± 5.3	−3.2 ± 1.1	−0.7 ± 0.6
∆Absolute muscle mass (Kg)	−0.0 ± 0.5	0.6 ± 0.5	0.5 ± 0.5	−2.7 ± 4.9
∆Relative muscle mass (%)	0.5 ± 0.4	0.1 ± 0.5	1.6 ± 0.5	−5.0 ± 6.4
∆Structured physical activity (min)	16.9 ± 47.0	−5.3 ± 61.7	−16.5 ± 25.4	16.9 ± 19.1
∆Total physical activity (min)	50.6 ± 102.1	−19.2 ± 80.3	−19.2 ± 28. 0	−23.5 ± 25.1

*BMI* body mass index

Statistically significant result between (1) No perception and perception of weight gain; (2) Perception of weight loss and perception of weight gain

The BMI Z-Score decreased with a statistical difference between the groups with no perception and those with a perception of weight gain (MD: 0.98, *p* = 0.002). Meanwhile, absolute fat mass showed a statistically significant difference between the groups with perceived weight loss and perceived weight gain (MD: 5.1 kg, *p* = 0.011) (Table 6).

### Longitudinal changes in anthropometrics, body composition and physical activity according to perceived versus real weight gain

With regard to body weight, there was a statistically significant difference between the groups with a correct perception of weight loss and weight gain (DM: 7.1 kg, *p* = 0.001), between the groups with correct and incorrect perception of weight gain (MD: 9.7 kg, *p* < 0.001), as well as between the groups with incorrect perception of weight gain and weight loss (MD: 7.3 kg, *p* < 0.001) (Table 7).

**Table 7:** Longitudinal changes in anthropometrics. body composition and physical activity according to perceived versus real weight gain

*Variables*	Correct perception of weight loss	Correct perception of weight increase	Wrong perception of weight increase	Wrong perception of weight loss
∆Height (cm)	0.6 ± 0.2	2.3 ± 0.4	1.3 ± 0.3	1.7 ± 0.4
∆Weight (kg)	**−1.5 ± 0.7 **^**(1)**^	**5.6 ± 0.6**^**(1) (2)**^	**−4.1 ± 0.5**^**(2) (3)**^	**3.2 ± 0.6 **^**(3)**^
∆BMI (kg/m^2^)	−0.78 ± 0.20	**1.23 ± 0.22**^**(2)**^	**−2.02 ± 0.26**^**(2) (3)**^	**0.59 ± 0.20**^**(3)**^
∆BMI Z-Score	−1.65 ± 0.06	**0.03 ± 0.05 **^**(2)**^	**−0.42 ± 0.05**^**(2) (3)**^	**0.00 ± 0.09 **^**(3)**^
∆Waist circumference (cm)	−4.7 ± 2.0	5.1 ± 2.1	−47.3 ± 43.3	3.8 ± 0.6
∆Hip perimeter (cm)	−0.3 ± 0.3	1.8 ± 1.4	−88.1 ± 58.8	−0.6 ± 3.9
∆Absolute fat mass (kg)	−1.7 ± 0.2	**2.5 ± 0.6**^**(2)**^	**−5.5 ± 1.5 **^**(2)**^	−0.4 ± 2.0
∆Relative fat mass (%)	−0.8 ± 0.7	**0.1 ± 0.7**^**(2)**^	**−4.1 ± 1.1 **^**(2)**^	−2.5 ± 2.4
∆Absolute muscle mass (Kg)	−0.5 ± 0.7	−3.5 ± 6.3	0.2 ± 0.5	1.4 ± 0.3
∆Relative muscle mass(%)	0.4 ± 0.4	−6.8 ± 8.1	1.9 ± 0.5	0.4 ± 0.5
∆Structured physical activity (min)	−55.0 ± 103.3	12.3 ± 19.3	−22.3 ± 24.2	53.0 ± 48.6
∆Total physical activity (min)	−98.3 ± 162.7	−38.0 ± 26.5	−23.5 ± 28.3	45.1 ± 49.1

*BMI* Body Mass Index

Statistically significant result between (1) Correct perception of loss and correct perception of increase; (2) Correct perception of increase and wrong perception of increase; (3) Wrong

perception of increase and wrong perception of loss

BMI variations and the BMI Z-score showed statistically significant differences between the groups with correct and incorrect perception of weight gain (DM ∆Weight: 3.25 kg/m^2^, *p* < 0.001; DM ∆BMI: 0.45, *p* < 0.001). In addition, there were also statistical differences in BMI (DM: 2.61 kg/m^2^, *p* < 0.001) and BMI Z-score (DM: 0.42, *p* < 0.001) between the groups with a misperception of weight gain and weight loss (Table 7).

As for the change in fat mass (absolute and relative), there were statistically significant differences between the groups with a correct perception of weight gain and those with a wrong perception of weight gain (DM: 8.0 kg, *p* < 0.001) (Table 7).

## Discussion

Obesity is one of the biggest challenges of the twenty-first century [15], with adolescence being a critical period in the development of this condition, partly associated with the numerous physiological and behavioral changes [6]. In addition, during adolescence, self-image has a marked relevance, a variable that can influence the course of this pathology [16].

There is no consensus in the literature about how (self) weight perception can influence weight development in adolescents with obesity. Thus, this study aimed to investigate the influence of weight perception on the evolution of body composition in adolescents with obesity followed in a Pediatric Obesity Clinic.

In this study, most adolescents (92.8%) of the participants showed a perception of weight evolution, revealing that most adolescents with obesity are aware of their body weight evolution, which may represent a positive starting point for therapeutic adherence. In fact, the literature indicates that the subjective perception of body evolution is a relevant predictor of behavioral change [17]. However, the study data revealed that perception alone is not enough; its accuracy is crucial to translate into adaptive behavior.

In terms of perceived versus actual weight gain, there was a high rate of incorrect perception of weight gain, with 55.8% of adolescents incorrectly perceiving their weight gain. This high percentage confirms that the perception of weight gain in adolescents with obesity is often distorted, even when they are under clinical supervision [18]. This incongruity can compromise the effects of educational and behavioral interventions.

In addition, the high prevalence (41.4%) of participants with an incorrect perception of weight gain has potential clinical implications and may indicate hypersensitivity to body image [19], and is associated with a greater risk of restrictive or inappropriate eating behaviours [20]. Finally, this may also lead to demotivation, hindering the continuity of the therapeutic plan.

The inaccurate perception of weight loss (14.4%) can lead to an increased risk of obesity in the long term, since the incorrect belief of weight loss can lead to a reduction in physical activity or less careful food intake. The literature argues that adolescents with an incorrect perception of weight loss are less likely to initiate or maintain behaviors of change, making it necessary to intervene in this type of cognitive error [21, 22].

On the other hand, the correct perception of weight gain can lead to greater involvement and adherence to goals and therapeutic plan, as long as there is adequate monitoring [23].

However, the low rate of correct perception of weight loss (3.9%) reveals a high discrepancy between reality and awareness of progress.

Finally, the data presented showed no statistically significant difference between sexes in the perception of weight gain, although the literature shows different sexual patterns [24]. The homogeneity of results between the sexes can be explained by the presence of obesity in the participants, which can reduce subjectivity in self-assessment [16].

As for the association between subjective perception of weight change, anthropometric measurements and physical activity, it was found that the group of participants with no perception of body change showed a significant increase in hip circumference, an objective measure of weight change. This finding once again reinforces the idea that many adolescents with obesity do not see themselves as such, and that acquiring competence is a fundamental step in inducing subsequent change [25].

The group of participants who perceived weight loss was associated with a real increase in BMI, as well as an increase in structured physical activity. Adolescents who perceive a weight gain tend to reinforce healthy habits, particularly physical activity [26], and this positive perception may be associated with positive reinforcement of healthy lifestyle measures, even if it is associated with an increase in BMI in the short term.

The perception of weight gain was associated with a real increase in BMI. The perception of negative weight gain can be accompanied by feelings of shame, leading to maladaptive eating behaviors and greater sedentary lifestyles [27, 28].

The group that had a correct perception of weight gain was also associated with a real increase in BMI, suggesting that awareness of the condition is a fundamental prerequisite for initiating meaningful behavioral change[25]. In this case, although in this short period these individuals had worse weight evolution, in the long term, self-awareness and behavioral change are associated with better results [29].

Finally, the incorrect perception of weight loss was correlated with a significant increase in BMI. In fact, the mistaken feeling of positive weight gain leads to a decrease in the stimulus to change harmful behaviors and may be associated with cognitive illusions or avoidance of discomfort [30].

As for anthropometric differences according to perceived weight loss, there were statistically significant differences between the group of participants with no perception of weight gain and with a perception of weight gain, as well as between the groups with a perception of weight loss and weight gain. These data support the hypothesis that different groups have unequal health literacy and body image sensitivity, both of which are fundamental for inducing behavioral change [25].

Finally, as expected, the differences in the evolution of weight were statistically significant between the group with a correct perception of weight loss and weight gain, as well as between the participants with an incorrect and correct perception of weight loss. The evolution of weight, BMI, BMI Z-Score and fat mass (absolute and relative) was significant among the group with the correct perception of weight gain and the wrong perception of weight gain. This may be explained by the fact that adolescents with greater body awareness are more likely to recognize real variations in weight and reinforce positive behaviors. In addition, the group that incorrectly perceived weight gain ended up reducing it, which may show body image disturbances associated with low self-esteem and distortion of weight perception.

This study presents some limitations that should be considered when interpreting the findings. For instance, its design limits the ability to infer causality between weight perception and subsequent changes in body composition. Although associations were identified, causal pathways cannot be established. In addition, the study used a convenience sample of adolescents followed in a specialized Adolescent Obesity Clinic, which may reduce the generalizability of the findings to broader adolescent populations, including those not receiving clinical care. The sample was further reduced by the requirement for valid longitudinal data, which, despite a good retention rate, may introduce selection bias. Another limitation associated with the setting may be the method used to assess weight perception through self-report during clinical interviews, which could lead to social desirability bias, recall bias, or difficulties in articulating one’s own body awareness. This limitation is particularly relevant given the psychological complexity of body image during adolescence. Also, the follow-up period was relatively short (median of 6 months) and not completely homogeneous (due to administrative issues). It can be argued that 6 months may not be sufficient to capture more gradual changes in body composition or the long-term implications of weight perception accuracy. Similarly, lifestyle behavior measures relied on interview-based assessments rather than objective tools (e.g., accelerometry), which may limit precision. Finally, the study did not account for psychological variables, which may mediate or moderate the relationship between weight perception and weight evolution. Thus, future research incorporating psychological constructs, such as body dissatisfaction, self-esteem, or readiness to change, would provide a more comprehensive understanding of the mechanisms involved.

Despite the acknowledged limitations, this study emphasizes the importance of accurate body weight perception in adolescents with obesity, as an inaccurate perception may negatively influence real weight changes and clinical outcomes. Accurate perception is a key factor in effective prevention and treatment strategies. Future research with larger samples and longer follow-ups, with the assessment of psychological constructs, is needed to better understand the impact of body weight perception on long-term weight trends and chronic health conditions in adolescents with obesity.

## Data Availability

No datasets were generated or analysed during the current study.

## Abbreviations

BMI: Body Mass Index
CAML: Centro Académico de Medicina de Lisboa
CI: Confidence Interval
MD: Mean Difference
ULSSM: Unidade Local de Saúde de Santa Maria
WHO: World Health Organization
